# Supplementary material for: Temporal dynamics of pro-inflammatory cytokines and serum corticosterone following acute sleep fragmentation in male mice
Source: PLoS One. 2023 Dec 14;18(12):e0288889. doi: 10.1371/journal.pone.0288889 (PMC10721077; doi:10.1371/journal.pone.0288889)
Supplement: Attachment — Submitted filename: Response to reviewers comments.docx [file pone.0288889.s001.docx]

**Responses in bold**

We note that Figure 1 in your submission contain copyrighted images.

**Thank you. We have made a new original figure to replace Figure 1.**

ACADEMIC EDITOR:

- please do follow directions from our reviewer to improve your study

**We believe that we have satisfactorily addressed the reviewer’s comments below.**

- could some of your results be explained by alteration in hormonal status affected by sleep fragmentation.

**This raises an interesting point. It is possible that sleep fragmentation could alter other hormones (besides glucocorticoids) that could affect inflammatory responses, e.g., androgens. We discuss this possibility in the discussion.**

Extend discussion to neuroinflammation and explain potential limitations and clinical benefits of your results more deeply.

**We have provided more info on neuroinflammation and also discuss potential limitations and clinical benefits in the discussion.**

Reviewer #1: This is a straightforward study that examines serum corticosterone and serum IL-6, as well as Il-1b mRNA and TNF-a mRNA) after experimental acute sleep fragmentation in male mice. The methods appear generally sound. However, I have some suggestions for improvement.

Major comments
- Title. I would suggest “Temporal dynamics of corticosterone and pro-inflammatory cytokines following acute sleep fragmentation in male mice” (or something similar).

**Thank you. We have made this change and have added “serum” in front of corticosterone.**

- General comment. Fewer abbreviations would make the text easier to read (e.g. can remove “SF” and “OSA”). Why not use “control” or “CON” instead of “NSF”?

**Thank you. We have cut down on the abbreviations in the text as recommended. We have spelled out OSA. We also have replaced “NSF” with “CON” throughout texts and figures. However, we believe that the abbreviation for SF should remain because we discuss it so much in this study. In the discussion, we attempt to remove SF and replace it with “sleep fragmentation” to make it more palatable to readers.**

- Methods. Why were only male mice used? This should be explained.

**We only used male mice to control for variation in immune/inflammatory responses that are well known to occur between the sexes. Adult female mice undergo a 4-day estrus cycle that may complicate their inflammatory responses to sleep fragmentation. Nonetheless, we are currently evaluating the temporal responses of female mice to sleep fragmentation and hope to compare them to male mice at a later date.**

- Methods. What was the body mass of the animals?

**We now report the average body mass of male mice used in the paper.**

- Methods. Please provide more information on the water, diet, and bedding. This is an important part of the ARRIVE guidelines.

**Thank you. We now report more information on the type of water used, the diet of the mice, and the type of bedding used.**

- Methods. For peripheral tissues, was the whole organ collected and used for analysis? How much tissue was collected for each brain region? How much tissue was used to measure gene expression?

**For peripheral tissues, a sub-sample of the whole organ was collected because the RNA extraction only permits less than 30 mg of tissue to be homogenized and measured for gene expression. Between 5-30 mg of brain tissue was collected for each brain region.**

- Methods. Why not measure serum IL-1b and TNF-a too? Why not measure IL-6 mRNA too? I did not understand the rationale for what was measured. This should be clearer.

**Thanks for your comments. We were limited in the amount of serum from each mouse, so were not able to assess more than one serum cytokine. The sandwich ELISA that we used is most sensitive to IL-6, so we made the decision to assess this pro-inflammatory cytokine in the serum. We were worried that IL-1b or TNF-a in the serum could be non-detectable (from prior experience). We would have liked to have measured IL-6 mRNA as well, but most of the previous studies in our lab have focused mostly on IL-1-beta and TNF-alpha gene expression, and the Taqman reagents/primers/probes are so expensive, we could not afford to measure another gene for this study.**

- Results. Figures for relative mRNA expression: Differing lowercase and uppercase letters are difficult to differentiate between 0, 1 and 2 hours.

**Thank you. We see what you are referring to. We have attempted to move those around slightly to make them more discernible.**

- Discussion. I am not sure *acute* sleep fragmentation reveals much about the effects of *chronic* apnea. This is worth a short paragraph in the Discussion.

**Thanks for pointing this out. We now provide a discussion on how the results are relevant to the etiology of chronic inflammatory diseases, like obstructive sleep apnea.**

Minor comments
- Figure 1. Consider indicating when the lights turn on (ZT0) and off (ZT12). Each rectangle during the duration of SF indicates 1 hour, but the two rectangles for the acclimation period represent two days (48 hours). Could clarify this in the legend.

**Thank you. We have now clarified this in the figure and the legend.**

- Figure 2B. The color and shape for NSF group (light blue circle) is different from the rest of the figures (dark blue triangle).

**Thanks. We have now fixed this.**

- Figure 4 legend. Typo: “PCF: prefrontal cortex”

**Thanks. This has been fixed**

- Results (line 270). Typo: “IL-1”

**Also fixed.**

- Discussion (lines 347-348). This sentence is a bit confusing. Consider rewording the sentence.

**We have now reworded the sentence. “IL-1β expression was significantly elevated in hypothalamus after 6 and 12 h of sleep fragmentation and in hippocampus after 24 h of sleep fragmentation; Fig 4, Table 1). A similar pattern for TNF-α gene expression was also observed occurring slightly later (Fig 4, Table 1). Acute sleep fragmentation did not affect TNF-α or IL-1β expression in prefrontal cortex (Fig 4, Table 1).”**
